# Pathological fracture following minimal trauma as the initial presentation of parathyroid carcinoma–associated hyperparathyroidism in a young man: a case report

**DOI:** 10.3389/fendo.2026.1785099

**Published:** 2026-05-08

**Authors:** Miljanka Vuksanović, Bojan Kovačević, Jelena Kuzmanović, Vladimir Vasić, Ognjen Krčmar, Milica Marjanović Petković

**Affiliations:** 1 Faculty of Medicine, University of Belgrade, Belgrade, Serbia; 2 Division of Endocrinology, Diabetes and Metabolic Disorders, Zvezdara University Medical Center, Belgrade, Serbia; 3 Faculty of Dentistry, University of Belgrade, Belgrade, Serbia; 4 Clinic for Surgery “Nikola Spasić”, Zvezdara University Medical Center, Belgrade, Serbia; 5 Department of Pathology, Zvezdara University Medical Center, Belgrade, Serbia; 6 Department of Urology, Zvezdara University Medical Center, Belgrade, Serbia; 7 Health Center Srbac, Srbac, Bosnia and Herzegovina

**Keywords:** bone mineral density, hypercalcemia, parathyroid carcinoma, parathyroid hormone, pathological fracture, primary hyperparathyroidism

## Abstract

**Background:**

Parathyroid carcinoma (PC) is a rare malignant endocrine tumor, accounting for approximately 0.005% of all malignancies and representing an uncommon cause of primary hyperparathyroidism. Excessive secretion of parathyroid hormone leads to severe hypercalcemia, resulting in multisystem complications, including skeletal fragility, nephrolithiasis, neuromuscular manifestations, and cardiovascular and neurocognitive disturbances. Preoperative diagnosis remains challenging, as PC frequently mimics benign parathyroid disease both clinically and radiologically.

**Case presentation:**

A 36-year-old man sustained a pathological fracture of the right radius during routine boxing training following minimal trauma. His medical history was notable for recurrent nephrolithiasis over the preceding six years, requiring multiple bilateral pyeloplasties, as well as several previously documented episodes of hypercalcemia of unknown etiology. Preoperative laboratory evaluation revealed severe hypercalcemia (4.0 mmol/L; reference range 2.2–2.7) and markedly elevated parathyroid hormone levels (2145 ng/L; reference range 15–65), consistent with primary hyperparathyroidism. Further endocrinological evaluation and imaging identified a parathyroid tumor. Surgical resection was performed, and histopathological examination confirmed the diagnosis of parathyroid carcinoma.

**Conclusion:**

This case highlights a pathological fracture following minimal trauma as the initial manifestation of parathyroid carcinoma–associated hyperparathyroidism in a young adult. It underscores the importance of considering endocrine etiologies in patients presenting with pathological fractures and unexplained hypercalcemia to enable timely diagnosis and appropriate management.

## 1 Introduction

Most parathyroid tumors are benign adenomas. Parathyroid carcinoma (PC) is an exceptionally rare malignant endocrine tumor, representing only 0.5–1% of cases of primary hyperparathyroidism (pHPT) (1). Excessive secretion of parathyroid hormone (PTH) leads to severe hypercalcemia, which may result in skeletal demineralization, muscle weakness, gastrointestinal disturbances, nephrolithiasis, and cardiovascular and neurocognitive complications.

Parathyroid carcinoma affects women and men equally and typically presents after the age of 40 (2). Preoperative diagnosis remains challenging because PC closely mimics benign parathyroid disease in both clinical presentation and imaging findings. The disease often follows a slow and indolent course, with morbidity primarily related to complications of hypercalcemia rather than local invasion or metastatic spread (3). However, none of these features are absolute, and atypical presentations may delay diagnosis and definitive treatment.

## 2 Case presentation

**Figure 1:**
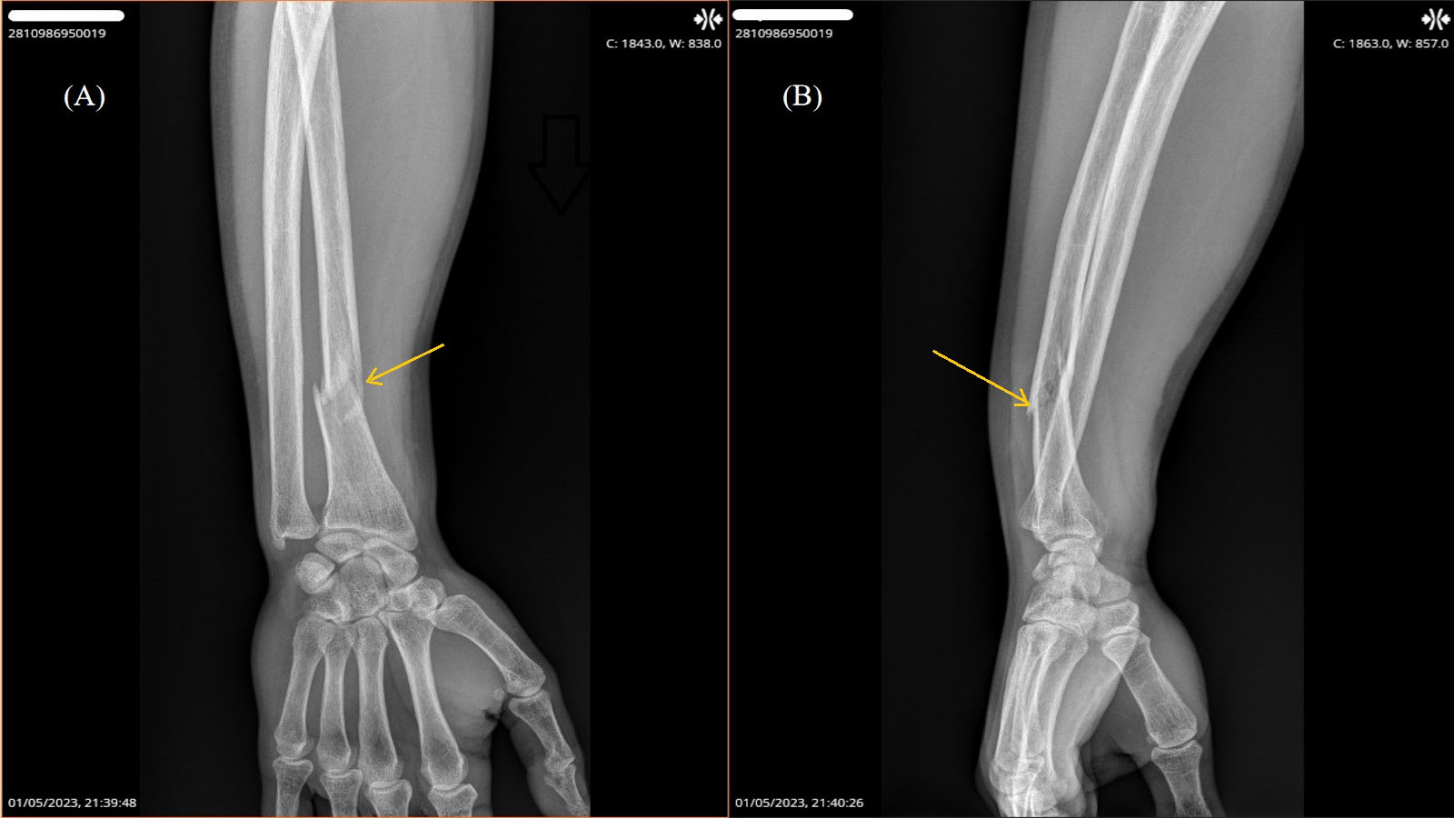
X-ray of the right radius showing a pathological fracture: **(A)** anteroposterior view; **(B)** lateral view.

A 36-year-old man sustained a fracture of the right radius (**Figure 1**) on May 1, 2023, during routine boxing training following minimal trauma. There was no history of high-energy injury. He was evaluated by an orthopedic surgeon, who recommended surgical fixation with osteosynthesis.

Preoperative laboratory evaluation revealed impaired renal function, with elevated creatinine (177 µmol/L; reference range 62–106) and urea (10.0 mmol/L; reference range 2.5–8.3), mild hyperkalemia (5.3 mmol/L; reference range 3.4–5.1), and marked hypercalcemia (4.0 mmol/L; reference range 2.2–2.7). Serum albumin (43 g/L; reference range 35–52) and phosphate levels (0.95 mmol/L; reference range 0.81–1.5) were within normal limits. Prior to surgery, the patient developed severe arterial hypertension (240/120 mmHg), prompting postponement of the orthopedic procedure and initiation of antihypertensive therapy with prazosin hydrochloride (2 mg). Electrocardiography showed sinus rhythm (70–75 bpm) with flattened T waves in lead D3 and no other significant abnormalities.

Abdominal and pelvic ultrasonography demonstrated advanced bilateral renal pathology. The right kidney measured 13.7cm, with grade III/IV hydronephrosis, pelvic dilatation up to 45mm, and multiple calculi. The left kidney measured 10.5cm, with a mildly dilated pyelocalyceal system and a 17-mm calculus in the lower pole. Bilateral parenchymal thinning and loss of corticomedullary differentiation were noted.

The patient**’**s medical history was significant for recurrent nephrolithiasis and bilateral ureteropelvic junction stenosis, for which he had undergone bilateral pyeloplasty five years earlier, as well as multiple double-J stent placements. Hypercalcemia had been documented as early as 2016; however, parathyroid hormone levels had not been assessed despite persistent renal calculi.

Measurement of serum PTH revealed a markedly elevated level of 2145 ng/L (reference range 15–65), strongly suggestive of primary hyperparathyroidism. The patient was referred to the endocrinology department for further evaluation (pre-hospital laboratory findings are summarized in **Table 1**). Vitamin D levels were not assessed preoperatively, as the diagnosis of primary hyperparathyroidism was evident based on markedly elevated PTH and severe hypercalcemia. However, assessment of vitamin D may provide additional information in the evaluation of calcium–phosphate metabolism. An extended endocrine evaluation was performed to exclude multiple endocrine neoplasia (MEN) types 1 and 2 and to assess the hypothalamic–pituitary–gonadal axis, given the presence of hypogonadism and the possibility of estrogen-secreting gonadal tumors contributing to skeletal fragility.

**Table 1:** Biochemical parameters, tumor markers, and hormonal profile at presentation and at 2-year follow-up.

Biochemical parameters				
Parameter	At presentation	2 years follow-up	Units	Reference range
Glucose	5.6	5.3	mmol/L	3.6 - 6.1
Uric acid	496	540	umol/L	208.0 - 428.0
Creatinine	202	292	umol/L	62.0 - 106.0
Urea	11.7	15.6	mmol/L	2.5 - 8.3
Potassium (K)	5.0	5.2	mmol/L	3.4 - 5.1
Sodium (Na)	140	141	mmol/L	136.0 - 146.0
Chloride (Cl)	110	114	mmol/L	101.0 - 109.0
Bicarbonate	23.0	22.2	mmol/L	21.0 – 31.0
Calcium–phosphate metabolism				
Calcium	3.6	2.3	mmol/L	2.2 - 2.7
Phosphorus	0.69	0.85	mmol/L	0.81 - 1.5
Magnesium	0.64	0.70	mmol/L	0.73 - 1.2
ALP	280	95	U/L	40.0 - 129.0
Liver and inflammatory parameters				
AST	17	28	U/L	0.0 - 40.0
ALT	30	42	U/L	0.0 - 41.0
GGT	19	31	U/L	0.0 - 60.0
CRP	1.7	2.0	mg/L	< 5.0
Tumor markers				
CEA	1.41	1.33	ng/mL	0.0 - 5.0
PSA	1.8	3.630	ng/mL	0.0 - 4.0
AFP	4.1	3.9	IU/mL	0.0 - 6.7
HCG	<0.1	<0.1	mIU/mL	<2.0
Hormonal profile				
PTH	1996.0	104.40	ng/L	15.0 - 65.0
TSH	0.848	0.903	mIU/L	0.27 - 4.2
free T4	13.02	14.8	pmol/L	12.0 - 22.0
Calcitonin	5.26	7.34	pg/mL	<18.9
Cortisol (8 AM)	396.6	227.7	nmol/L	171.0 - 536.0
ACTH (8 AM)	36.72	27.6	pg/mL	7.0 - 63.3
Testosterone	14.65	13.060	nmol/L	9.9 - 27.8
Prolactin (8 AM)	365	288	µIU/mL	98.0 - 456.0
FSH	4.0	5.27	IU/L	1.5 - 12.4
LH	13.2	11.6	IU/L	1.7 - 8.6
Estradiol	18.35	62.12	pmol/L	28.0 - 156.0

AST, aspartate aminotransferase; ALT, alanine aminotransferase; ALP, Alkaline phosphatase; GGT, gamma-glutamyl transferase; CRP, C-reactive protein; CEA, carcinoembryonic antigen; PSA: prostate-specific antigen; AFP: alpha-fetoprotein; HCG, Human Chorionic Gonadotropin; PTH, Parathyroid hormone; TSH, Thyroid Stimulating hormone; CORT, cortisol; ACTH, Adrenocorticotropic hormone; FSH, Follicle-Stimulating Hormone; LH, luteinizing hormone.

**Figure 2:**
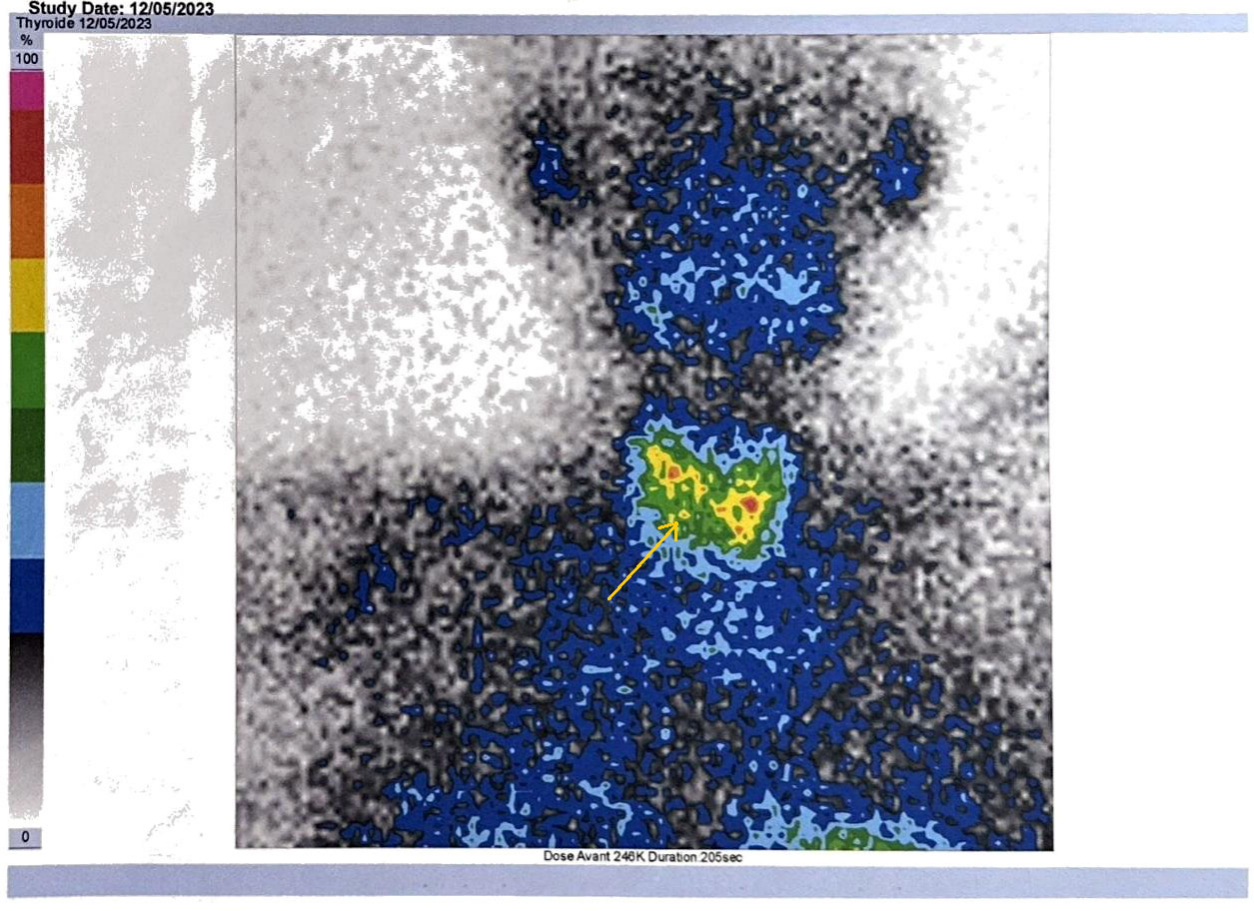
Parathyroid scintigraphy demonstrating a hyperfunctioning parathyroid gland.

Immediate treatment of hypercalcemia included intravenous rehydration and administration of zoledronic acid. After normalization of serum calcium (2.4 mmol/L), osteosynthesis of the right radius was successfully performed. Parathyroid scintigraphy was performed following the initial laboratory evaluation and prior to definitive surgical management, demonstrating a hyperfunctioning parathyroid gland (**Figure 2**). Subsequent contrast-enhanced computed tomography of the neck revealed a 26 × 20 × 32mm soft-tissue lesion adjacent to the caudal pole of the left thyroid lobe, suspicious for an enlarged and pathologically altered left superior parathyroid gland (**Supplementary Figure 1**). Several small cervical lymph nodes were noted bilaterally, without radiologic features of metastatic disease. Imaging of the chest and abdomen showed no evidence of distant metastases.

**Figure 3:**
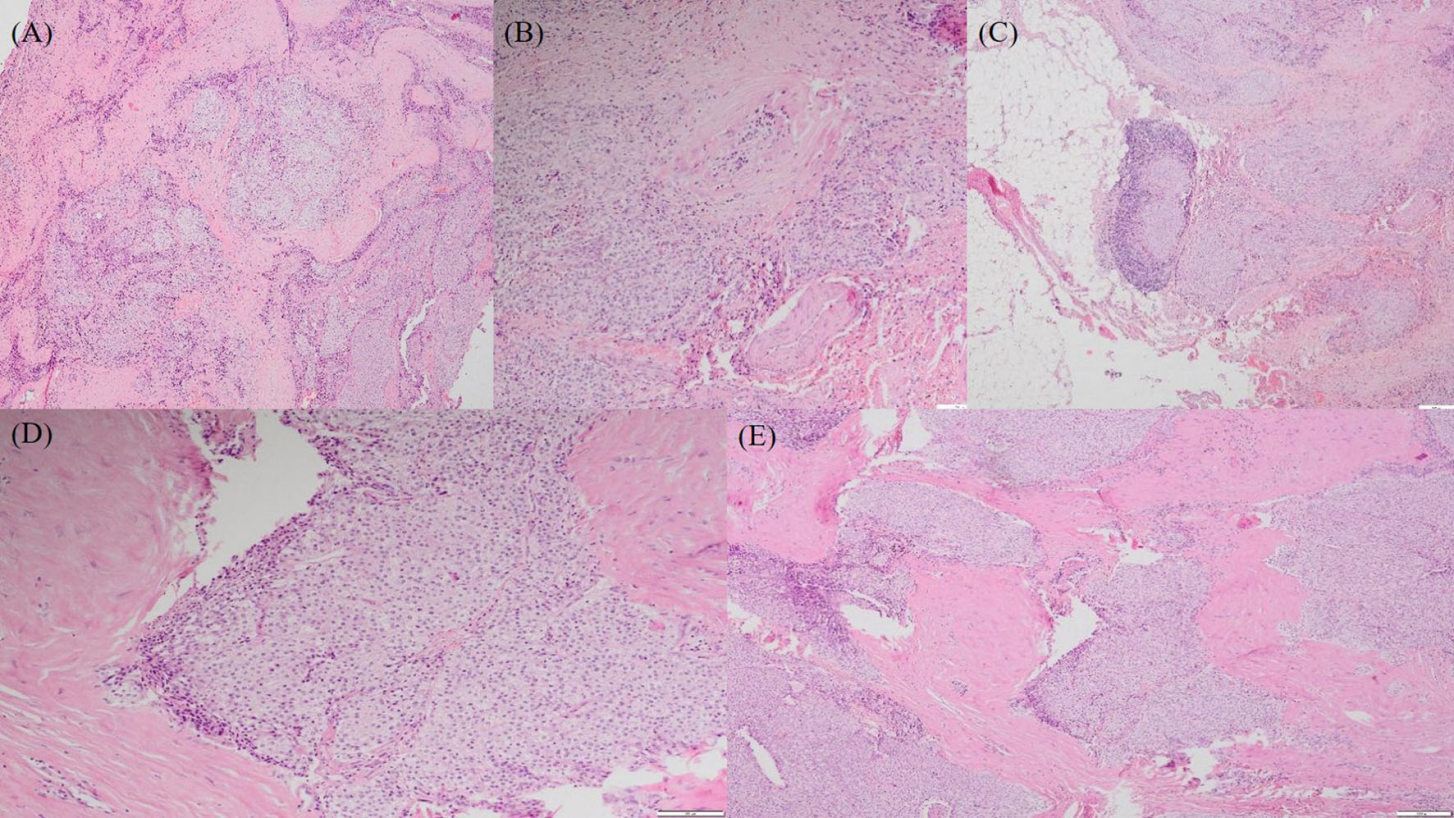
Microscopic features of the left superior parathyroid carcinoma (hematoxylin–eosin staining) **(A, B)** Chief cells with an increased nucleocytoplasmic ratio at low and intermediate magnification. **(C)** Tumor demarcation by broad fibrous connective tissue bands. **(D)** Capsular invasion. **(E)** Invasion of adjacent skeletal muscle.

Based on clinical, biochemical, and imaging findings, a parathyroid tumor was suspected. The patient underwent en bloc surgical resection, including removal of the left superior parathyroid gland and the left thyroid lobe. Intraoperatively, a firm tumor measuring approximately 3cm and infiltrating the esophageal muscular layer and the left recurrent laryngeal nerve was identified (**Supplementary Figures S2**, **S3**). Histopathological examination demonstrated a neoplasm composed predominantly of chief cells with an increased nucleocytoplasmic ratio, broad fibrous bands, capsular invasion, and invasion into adjacent skeletal muscle (**Figure 3**), confirming the diagnosis of parathyroid carcinoma.

Bone mineral density assessment using dual-energy X-ray absorptiometry revealed severe osteoporosis, with Z-scores of −3.2 at the lumbar spine, −3.2 at the femoral neck, and −4.9 at the distal radius (**Supplementary Figure 4**). Osteoporosis-specific therapy was not initiated prior to surgery, as the primary therapeutic approach was directed toward correction of the underlying hyperparathyroidism.

The postoperative course was uneventful. Serum calcium and PTH levels normalized (Ca 2.1 mmol/L; PTH 20 ng/L), and the patient was discharged on postoperative day 7. Genetic testing identified a pathogenic monoallelic mutation in the CDC73 gene, consistent with hereditary hyperparathyroidism and an increased risk of parathyroid carcinoma.

At 12-month follow-up, the patient remained asymptomatic with normal serum calcium levels. At 2-year follow-up, laboratory findings remained within normal ranges. Dual-energy X-ray absorptiometry demonstrated improvement in bone mineral density (**Supplementary Figure 5**), and whole-body computed tomography showed no evidence of recurrence or metastatic disease (**Supplementary Figure 6**). A timeline summarizing the key clinical events is presented in **Table 2**.

**Table 2:** Timeline of clinical events, diagnostic evaluation, and treatment.

Time	Event
2016	Hypercalcemia first noted
2018–2023	Recurrent nephrolithiasis
May 2023	Pathological fracture
Pre-op	Severe hypercalcemia, ↑PTH
Surgery	Parathyroid tumor resection
12 months	Normal Ca
2 years	No recurrence, BMD improved

PTH, Parathyroid hormone; Ca, calcium; BMD, Bone mineral density.

## 3 Discussion

Parathyroid carcinoma is a rare cause of primary hyperparathyroidism and remains a diagnostic challenge. Distinguishing PC from benign parathyroid adenoma preoperatively is difficult, as clinical presentation and imaging findings frequently overlap (4). Consequently, the diagnosis is often established only after histopathological examination, as in the present case.

Most parathyroid carcinomas are hormonally functional and present with severe manifestations of hypercalcemia, including nephrolithiasis, bone disease, and neuromuscular symptoms (5). Skeletal involvement, including bone pain, osteitis fibrosa cystica, and pathological fractures, has been reported and may represent the initial manifestation of disease (6). In our patient, long-standing renal complications preceded the diagnosis by several years, underscoring the potential for delayed recognition.

Markedly elevated serum calcium and PTH levels, tumor size greater than 3 cm, and severe skeletal and renal involvement are clinical features that should raise suspicion for PC (7–10). Our patient fulfilled all proposed clinical criteria, including young age, extreme biochemical abnormalities, and combined bone and kidney disease. In this case, osteoporosis-specific therapy was not initiated prior to surgery, as correction of the underlying hyperparathyroidism represents the primary therapeutic approach and is often associated with significant improvement in bone mineral density following successful surgical treatment. Additionally, an extended endocrine evaluation, including assessment of the hypothalamic–pituitary–gonadal axis, was performed to exclude secondary causes of bone fragility, which is particularly relevant in younger patients.

Genetic factors play a role in a subset of cases. Loss-of-function mutations in the CDC73 tumor suppressor gene are associated with hereditary hyperparathyroidism and parathyroid carcinoma, particularly in hyperparathyroidism–jaw tumor syndrome and familial isolated hyperparathyroidism (11, 12). Identification of a pathogenic CDC73 mutation in this patient has important implications for long-term surveillance and family screening.

At the time of the initial clinical evaluation, genetic testing results were not yet available, as the analysis had been performed in an external institution and provided subsequently by the patient. Therefore, targeted assessment for features suggestive of hyperparathyroidism–jaw tumor (HPT-JT) syndrome, including jaw imaging, was not performed initially (12). Following identification of the CDC73 pathogenic variant, the patient was advised to undergo further evaluation, including radiological assessment of the jaw. However, this has not yet been completed.

Given the known association between CDC73-related conditions and renal tumors (12), renal evaluation was carefully performed. The patient underwent multiple contrast-enhanced abdominal CT scans both prior to and during the index hospitalization, none of which revealed evidence of renal neoplasia. In addition, urological assessment excluded renal malignancy. Furthermore, during follow-up, a whole-body CT performed two years after surgery showed no signs of renal tumor development, supporting the absence of renal involvement to date.

Complete en bloc resection at initial surgery offers the best chance for cure and is associated with improved long-term survival (13). Recurrence is common when complete resection is not achieved, typically occurring within 2–5 years (14, 15). In this case, early definitive surgery and close follow-up have thus far resulted in sustained remission without evidence of recurrence.

Given the presence of a CDC73 pathogenic variant, lifelong surveillance is warranted due to the risk of recurrence and the development of associated neoplasms. In this context, biochemical monitoring is recommended, including serum calcium and PTH measurements every 3–6 months during the first two years following surgery, and annually thereafter. Neck imaging (ultrasound, with additional cross-sectional imaging such as CT or MRI if clinically indicated) should be performed in cases of biochemical or clinical suspicion of recurrence. Considering the association with hyperparathyroidism–jaw tumor (HPT-JT) syndrome, evaluation for jaw lesions is advised, including baseline dental examination and orthopantomography, with further imaging if abnormalities are detected. In light of the known association between CDC73 mutations and renal tumors, baseline renal imaging is recommended, followed by periodic surveillance (e.g., every 1–2 years), although standardized intervals have not yet been clearly established.

This structured and individualized follow-up approach is essential for early detection of both disease recurrence and associated neoplasms. Given the absence of standardized surveillance protocols for CDC73 mutation carriers, multidisciplinary management remains crucial (8, 12).

## 4 Conclusion

Parathyroid carcinoma should be suspected in young patients presenting with severe hypercalcemia, markedly elevated PTH levels, and combined skeletal and renal manifestations, including pathological fractures. Early recognition and complete en bloc surgical resection, supported by a multidisciplinary approach, are critical for optimal outcomes and long-term disease control.

The patient reported significant improvement in overall well-being following surgical treatment. He noted resolution of previous symptoms, including fatigue and recurrent renal issues, and expressed satisfaction with the outcome of treatment and follow-up care.

## Data Availability

The original contributions presented in the study are included in the article/**Supplementary Material**. Further inquiries can be directed to the corresponding author.
