# Supplementary material for: Pathological fracture following minimal trauma as the initial presentation of parathyroid carcinoma–associated hyperparathyroidism in a young man: a case report
Source: Front Endocrinol (Lausanne). 2026 May 8;17:1785099. doi: 10.3389/fendo.2026.1785099 (PMC13193886; doi:10.3389/fendo.2026.1785099)

**Supplementary Figures:**

Supplementary Fig. S1 Neck CT scan demonstrating marked enlargement of the left superior parathyroid gland.


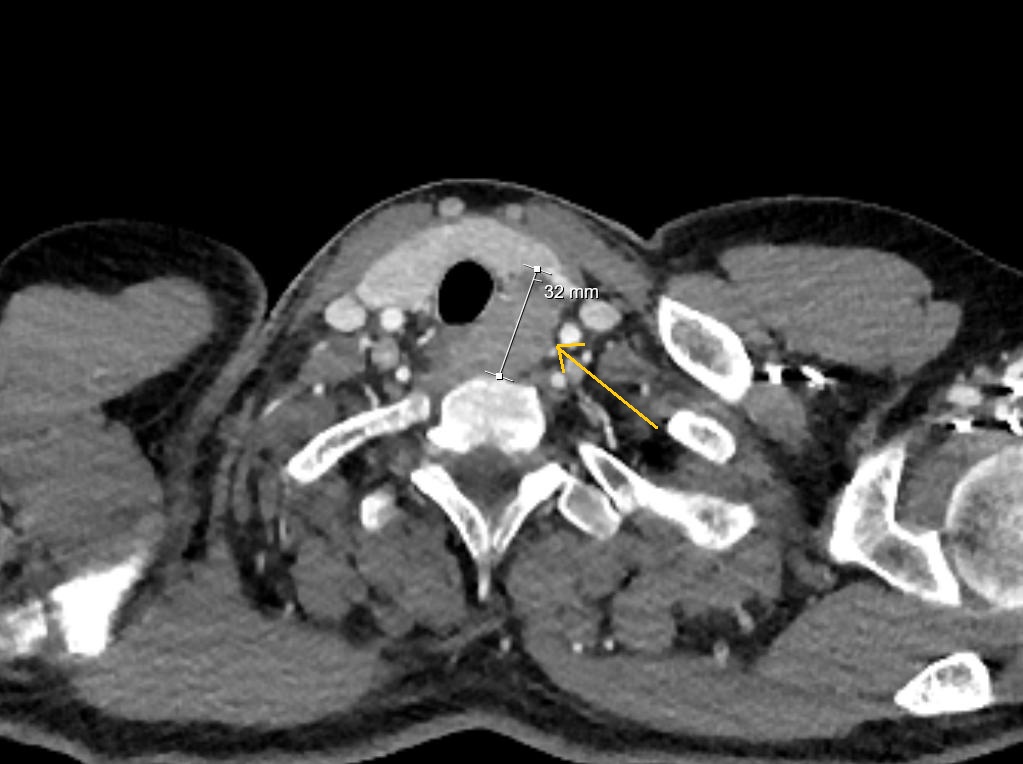


Supplementary Fig. S2 Intraoperative view of the parathyroid tumor.


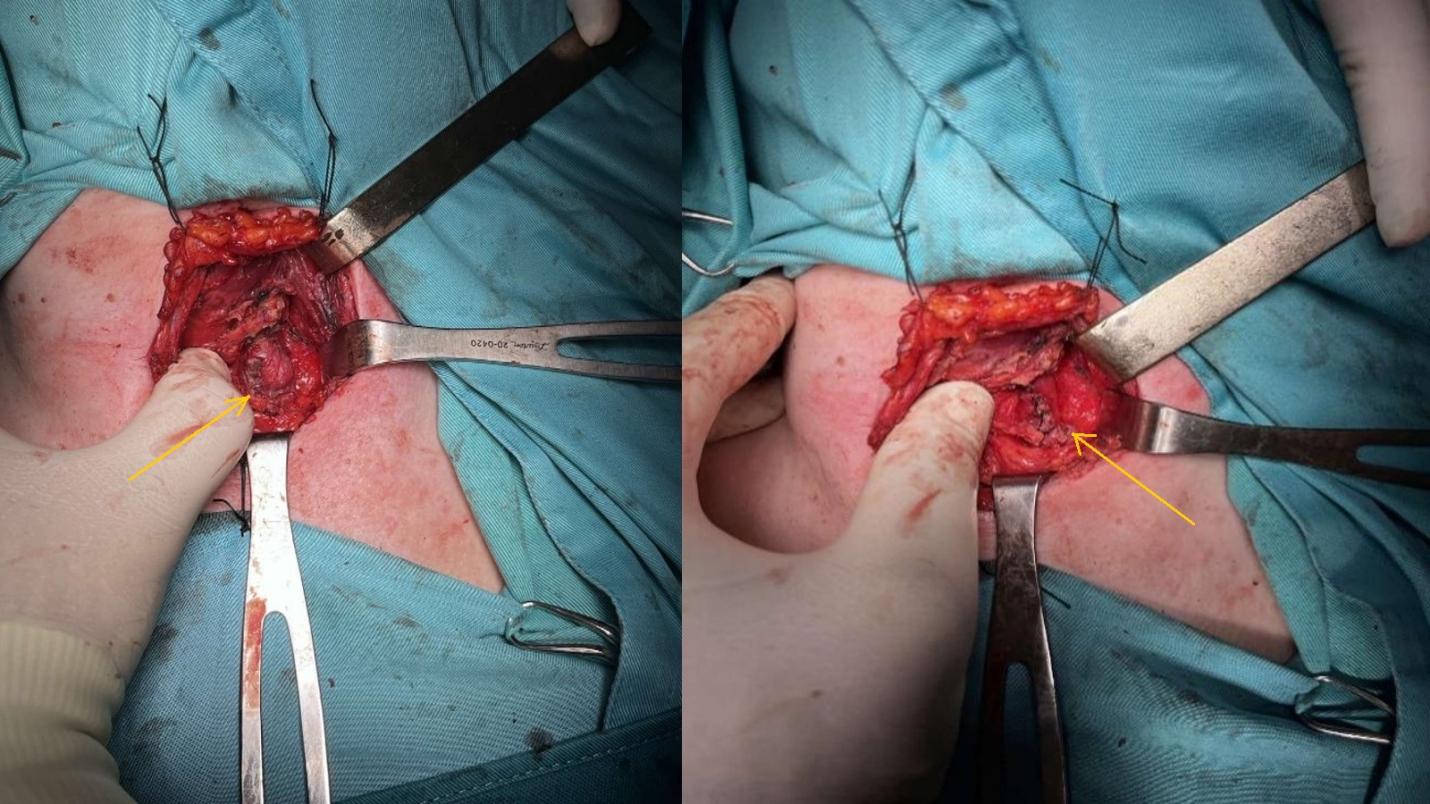


Supplementary Fig. S3 Macroscopic appearance of the parathyroid tumor.


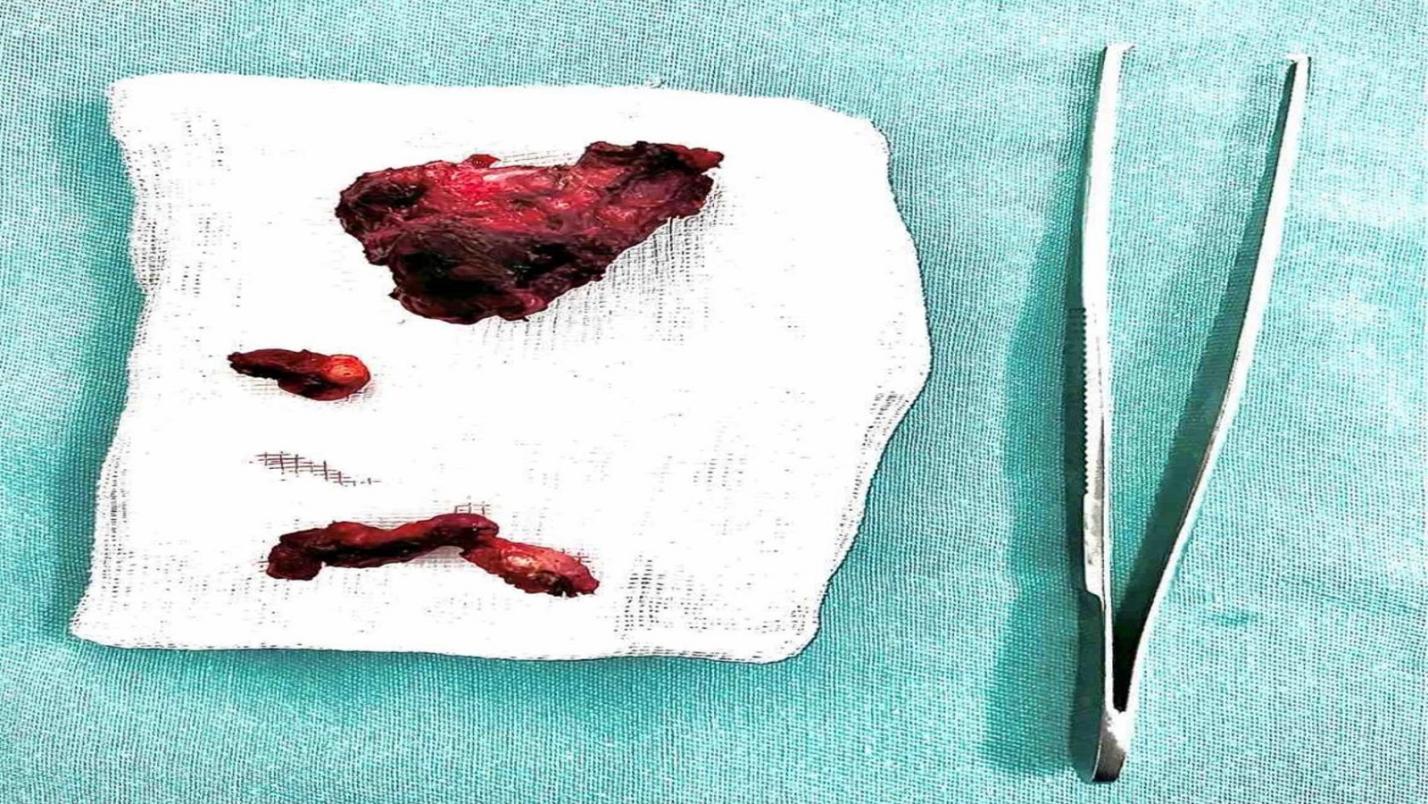


Supplementary Fig. S4 Dual-energy X-ray absorptiometry (DXA) of the lumbar spine (A), femoral neck (B), and distal radius (C) at baseline.


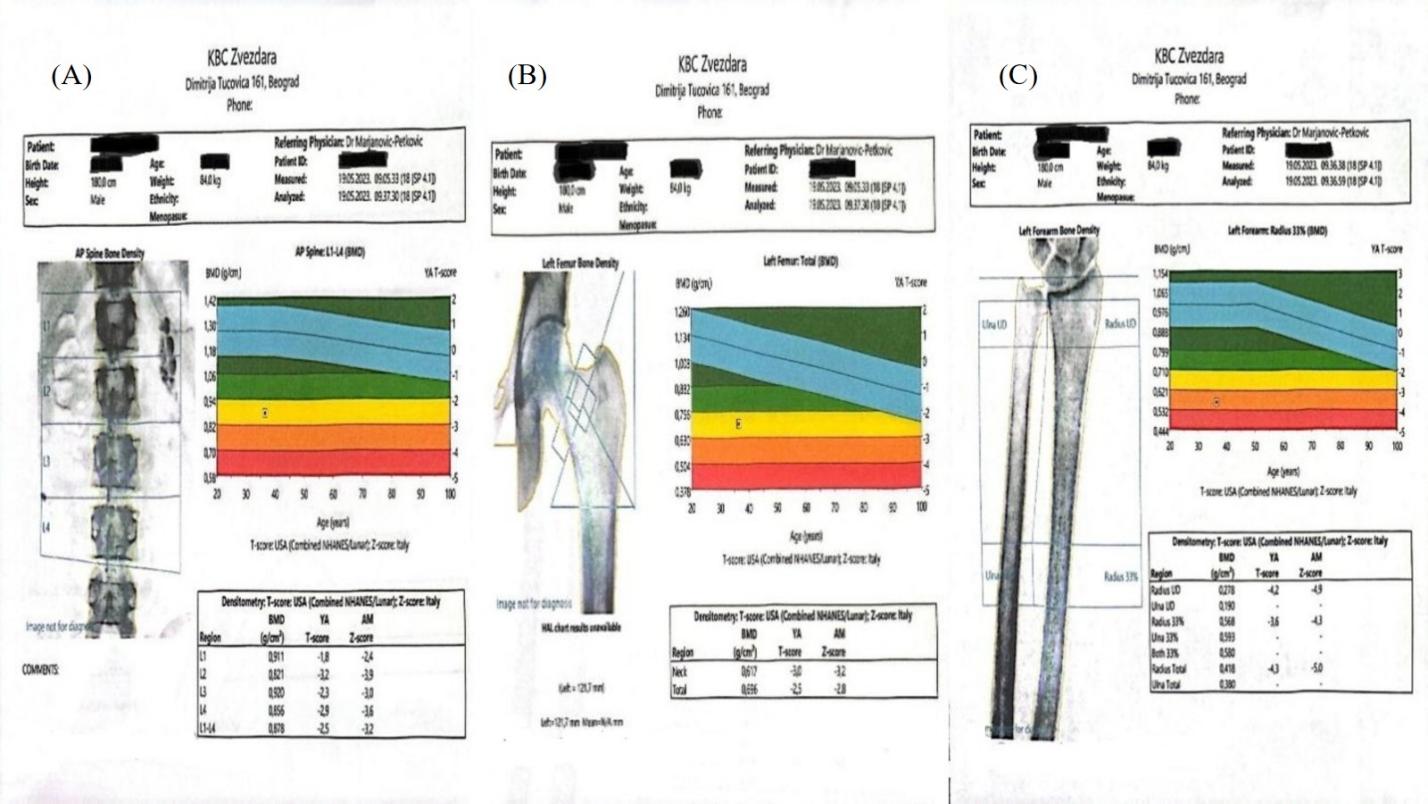


Supplementary Fig. S5 Follow-up dual-energy X-ray absorptiometry (DXA) of the lumbar spine (A), femoral neck (B), and distal radius (C) after 2 years.


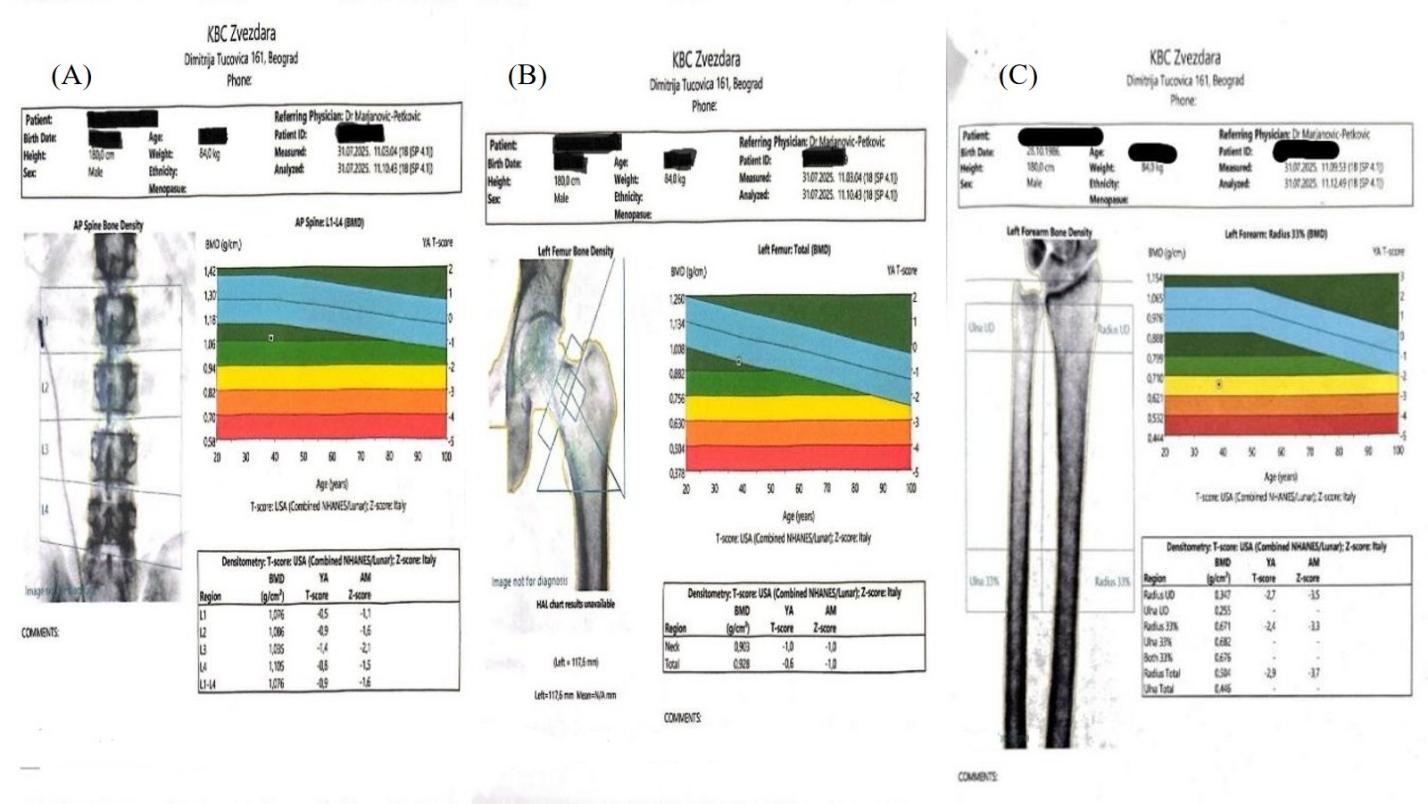


**Supplementary Fig. S6.** Contrast-enhanced CT scan of the neck at 2-year follow-up showing no evidence of local recurrence.


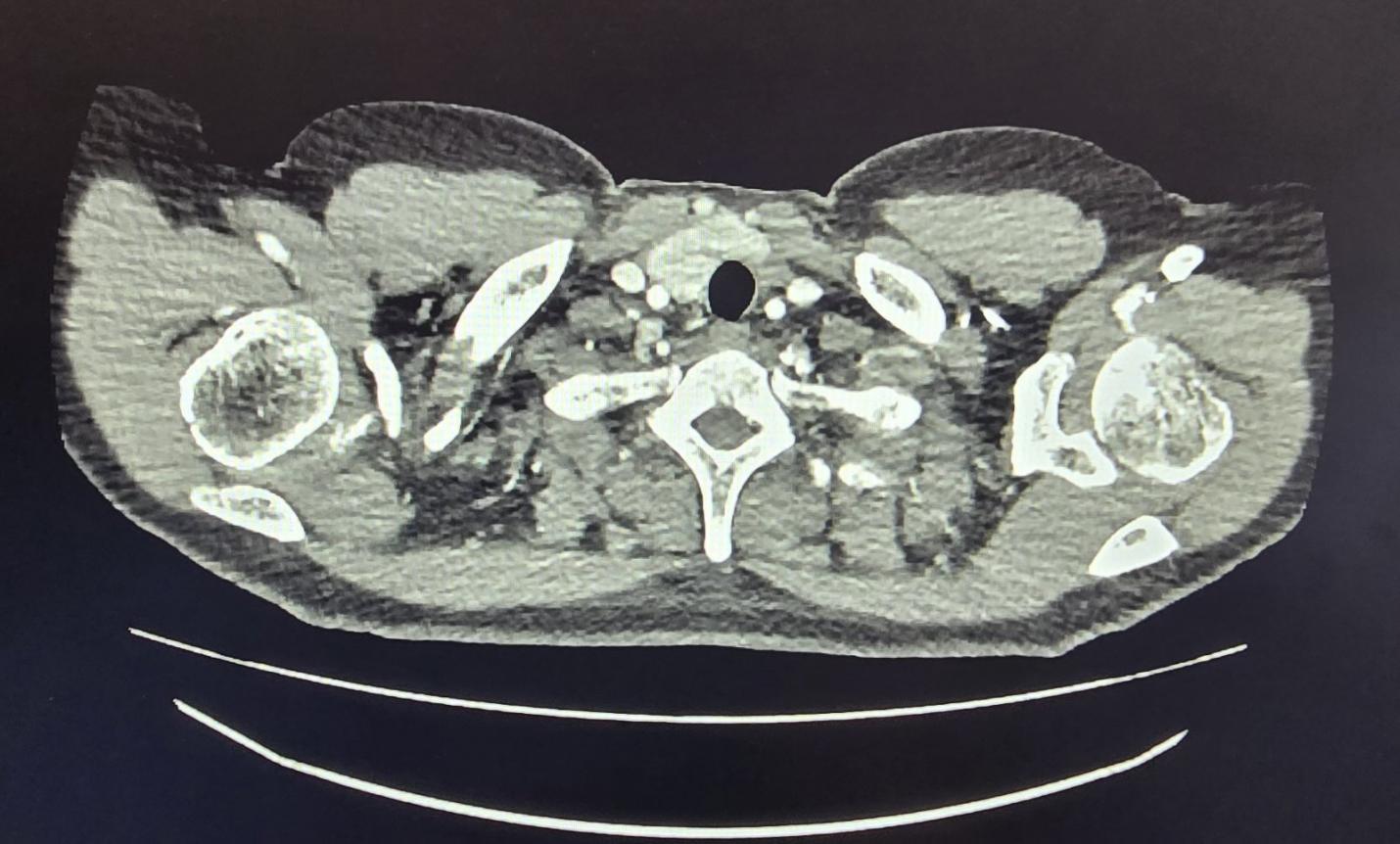

Supplement: Supplementary file 1 [file DataSheet1.docx]
