# Supplementary material for: Pathological fracture following minimal trauma as the initial presentation of parathyroid carcinoma–associated hyperparathyroidism in a young man: a case report
Source: Front Endocrinol (Lausanne). 2026 May 8;17:1785099. doi: 10.3389/fendo.2026.1785099 (PMC13193886; doi:10.3389/fendo.2026.1785099)
Supplement: Supplementary file 2 [file DataSheet2.docx]

## CARE Checklist

**Manuscript title:**
Pathological Fracture Following Minimal Trauma as the Initial Presentation of Parathyroid Carcinoma–Associated Hyperparathyroidism in a Young Man: A Case Report

| **Section** | **Item No.** | **Checklist Item** | **Yes/No** | **Reported on Page/Section** |
| --- | --- | --- | --- | --- |
| **Title** | 1 | The title includes the words “case report” and the phenomenon of greatest interest | Yes | Title page |
| **Key Words** | 2 | Key words identify diagnoses or interventions | Yes | Abstract – Keywords |
| **Abstract** | 3a | Introduction – What is unique about this case? | Yes | Abstract – Background |
|  | 3b | Main symptoms and clinical findings | Yes | Abstract – Case Presentation |
|  | 3c | Main diagnoses, interventions, and outcomes | Yes | Abstract – Case Presentation |
|  | 3d | Conclusion – What are the main lessons learned? | Yes | Abstract – Conclusion |
| **Introduction** | 4 | One or two paragraphs summarizing why this case is unique | Yes | Introduction |
| **Patient Information** | 5a | De-identified demographic information | Yes | Case Presentation |
|  | 5b | Main complaints and symptoms | Yes | Case Presentation |
|  | 5c | Relevant medical, family, and psychosocial history | Yes | Case Presentation |
| **Clinical Findings** | 6 | Physical examination and clinically relevant findings | Yes | Case Presentation |
| **Timeline** | 7 | Timeline of key events | Yes | Case Presentation (narrative) |
| **Diagnostic Assessment** | 8a | Diagnostic methods (laboratory tests, imaging, pathology) | Yes | Case Presentation |
|  | 8b | Diagnostic challenges | Yes | Case Presentation |
|  | 8c | Diagnostic reasoning and differential diagnoses | Yes | Case Presentation |
|  | 8d | Prognostic characteristics (e.g., staging, genetics) | Yes | Case Presentation |
| **Therapeutic Interventions** | 9a | Types of treatment (medical, surgical) | Yes | Case Presentation |
|  | 9b | Administration details (dose, duration) | Yes | Case Presentation |
|  | 9c | Changes in therapeutic interventions | Yes | Case Presentation |
| **Follow-up and Outcomes** | 10a | Clinician- and patient-assessed outcomes | Yes | Case Presentation |
|  | 10b | Follow-up tests and results | Yes | Case Presentation |
|  | 10c | Adverse or unanticipated events | No | Not observed |
| **Discussion** | 11a | Strengths and limitations of the case | Yes | Discussion |
|  | 11b | Comparison with literature | Yes | Discussion |
|  | 11c | Rationale for conclusions | Yes | Discussion |
| **Patient Perspective** | 12 | Patient perspective included | No | Not applicable |
| **Informed Consent** | 13 | Written informed consent obtained | Yes | Ethics Statement |
